# Research Progress on Nanomaterials in SPR Sensors

**DOI:** 10.3390/nano15241847

**Published:** 2025-12-08

**Authors:** Hongji Zhang, Zhe Gao, Yulin Zhang, Runze Hou, Haoran Zhang, Ziqi Yan, Jiazhen Tian, Pengcheng Tao, Xinlei Zhou

**Affiliations:** School of Optoelectronic Engineering and Instrumentation Science, Dalian University of Technology, Dalian 116024, China; zhanghongji@mail.dlut.edu.cn (H.Z.); 993870445@mail.dlut.edu.cn (Z.G.); zhangyulin@mail.dlut.edu.cn (Y.Z.); 201992239@mail.dlut.edu.cn (R.H.); 95763100@mail.dlut.edu.cn (H.Z.); 18617605338@mail.dlut.edu.cn (Z.Y.); tianjiazhen@mail.dlut.edu.cn (J.T.); pctao@dlut.edu.cn (P.T.)

**Keywords:** SPR sensor, nanomaterials, nanomaterial enhancement, biomolecular detection

## Abstract

While surface plasmon resonance (SPR) sensors serve as vital tools for biomolecular detection; conventional versions suffer from inherent limitations, including confined localized electromagnetic fields and inadequate sensitivity for detecting low-abundance analytes. Consequently, this paper reviews the progress of research in nanomaterial-enhanced SPR sensors to address these challenges. Initially, the review elaborates on the sensing principles and signal modulation strategies of SPR sensors. It systematically analyzes the enhancement mechanisms of noble metal nanoparticles (ranging from spherical 0D to advanced anisotropic 1D/2D nanostructures), magnetic nanoparticles (MNPs), and two-dimensional (2D) nanomaterials, alongside their applications in the detection of small molecules, nucleic acids, and biomacromolecules. Crucially, this review provides a comparative benchmarking of these materials, evaluating their trade-offs between sensitivity enhancement and practical stability. Furthermore, it identifies critical bottlenecks in industrialization, specifically addressing environmental challenges such as thermal cross-sensitivity and oxidative degradation, alongside issues of reproducibility and standardization. Finally, future research directions are proposed, including developing novel nanomaterials, exploring low-cost alternatives, and constructing flexible wearable sensing systems.

## 1. Introduction

Surface plasmon resonance (SPR) sensors are advanced optical devices extensively utilized for the label-free, real-time monitoring of biomolecular interactions, such as antibody–antigen binding and protein-DNA hybridization. Central to their operation are surface plasmon polaritons (SPPs), which are collective oscillations of free electrons propagating along the metal-dielectric interface. These SPPs are highly sensitive to changes in the refractive index near the sensor surface, allowing for precise detection [1]. However, traditional SPR sensors use metal films to excite surface plasmon polaritons (SPPs), which leads to limitations such as confined local electromagnetic field intensity and insufficient sensitivity for low-abundance analytes (target molecules present at trace concentrations, often in the femtomolar or picomolar range) [2]. These constraints hinder their applications in complex samples, such as clinical serum and environmental matrices [3].

Briefly, the SPR phenomenon occurs when the momentum of incident light matches that of the SPPs, resulting in a dip in reflected light intensity [1]. To address current limitations, breakthroughs in nanomaterial technology have provided key solutions: (i) noble metal nanoparticles (AuNPs, AgNPs) exhibit a localized surface plasmon resonance (LSPR) effect, enabling signal amplification; (ii) magnetic nanoparticles (e.g., Fe_3_O_4_) possess targeted enrichment capabilities, facilitating the separation of analytes from complex matrices; and (iii) 2D nanomaterials (e.g., graphene, MoS_2_) offer interface optimization and tunable optoelectronic properties, enhancing sensor stability and specificity [4,5].

This paper systematically reviews the fundamental principles and signal modulation methods of SPR sensors and thoroughly analyzes the enhancement mechanisms of three core nanomaterial categories, with a specific focus on the evolving role of anisotropic plasmonic structures. Unlike previous reviews, this work provides a critical comparative analysis of these materials, evaluating their performance benchmarks and stability limitations. It further explores integration trends with intelligent algorithms, multimodal detection, and microfluidic technologies, and summarizes current bottlenecks regarding real-time environmental stability (e.g., temperature and oxidation effects) and clinical translation [4]. It aims to provide theoretical references and design principles for optimizing SPR sensor performance and accelerating their practical application.

## 2. Fundamentals of SPR Sensors

### 2.1. Evolution of SPR Sensors

The development of SPR sensors spans over a century, with key milestones shaping their current form. In 1902, Wood [6] first observed the surface plasmon resonance phenomenon in optical experiments, laying the experimental foundation for SPR technology. In 1941, Fano proposed a theoretical interpretation for the SPR phenomenon [7], clarifying its physical nature. In 1959, Powell and Swan experimentally validated Ritchie’s theory: when high-energy electrons pass through a metal film, energy loss peaks occur at the bulk plasmon frequency and lower frequencies, attributed to the metal film interface [8]. In 1968, Otto experimentally verified the SPR theory and proposed a prism-type SPR sensing structure. Later that year, Kretschmann et al. [9] optimized Otto’s design to develop a novel prism-coupled SPR configuration, which remains widely used today.

However, the evolution of SPR technology has extended significantly beyond this conventional Kretschmann configuration to address limitations in sensitivity and integration. To overcome the bulkiness of prism-based setups, recent advancements have focused on optical fiber and planar waveguide-based SPR sensors. Specifically, optical fiber configurations enable remote in vivo monitoring capabilities, while planar waveguide integration facilitates microfabrication and high-throughput multichannel sensing. Furthermore, to address the broad spectral linewidths typical of traditional localized surface plasmons (LSPs), researchers have exploited diffractive coupling in periodic nanoparticle arrays to generate Surface Lattice Resonances (SLRs). These SLRs produce extremely narrow resonances (down to 1–2 nm) with high quality factors, significantly enhancing detection resolution. Correspondingly, these innovations have expanded the application scope of SPR sensors beyond biomedical research to diverse fields, including environmental monitoring (e.g., heavy metals) and food safety. Concurrently, the integration of novel materials, particularly 2D nanomaterials like graphene and transition metal dichalcogenides (TMDCs), has emerged as a critical strategy. Beyond serving as a protective layer to prevent oxidation, graphene plays a vital role in amplifying sensitivity by providing an extremely high surface-to-volume ratio for efficient biomolecular adsorption, thus addressing the demand for ultra-sensitive detection in complex matrices [10].

These evolutionary trends towards diversified structures and advanced materials are well-exemplified by recent research progress.

In 2011, Nunzio et al. [11] demonstrated the integration of SPR in waveguides by developing a low-cost sensor based on plastic optical fibers (POFs), where a thin gold film was deposited over a photoresist buffer layer on the exposed fiber core. This configuration expanded the detectable refractive index range to 1.332–1.418 and significantly improved the signal-to-noise ratio compared to buffer-free geometries, highlighting the efficacy of waveguide-integrated platforms for accessible biosensing applications [10].

In 2017, Saad Y et al. applied silver-coated optical fiber SPR sensors for chemical and biological applications [12], integrating a graphene protective layer to improve sensor stability. Concurrently, Wei W et al. modified graphene on plastic-coated quartz fiber SPR sensors [13], increasing the refractive index sensitivity from 2869 nm/RIU to 6500 nm/RIU—confirming the enhancement effect of 2D nanomaterials; Kumar et al. designed a Kretschmann-configuration biosensor for non-invasive urine glucose detection [14]. This approach utilizes various semiconductors combined with bimetallic layers and two-dimensional nanomaterials like black phosphorus and graphene to enhance biosensor sensitivity, demonstrating the practical application of graphene and black phosphorus in biosensing.

In 2022, Mohamed et al. designed a multifunctional SPR biosensor based on bimetallic films (Ag/Al_2_O_3_), BaTiO_3_ nanostructures, and 2D nanomaterials (Ti_3_C_2_T_x_, black phosphorus (BP) and BlueP/MoS_2_ heterostructures). This design achieved a maximum angular sensitivity of 504 °/RIU, representing a 320% improvement over existing 2D nanomaterial-integrated SPR sensors. Specifically, BaTiO_3_, Ti_3_C_2_T_x_, BP and BlueP/MoS_2_ nanostructures enhanced angular sensitivity by 120%, 60%, 290%, and 80%, respectively, while ensuring high-quality SPR curves [15].

In 2025, Guo et al. proposed a silver/graphene composite thin-film microstructured optical fiber sensor [16]. After graphene modification, the silver-plated sensor achieved a sensitivity of 2916.8 nm/RIU [16]. In the same year, Mst. Rokeya Khatun et al. designed a photonic crystal fiber (PCF)-SPR biosensor integrated with machine learning (ML) and artificial intelligence (AI), achieving a breakthrough in maximum wavelength sensitivity beyond traditional limits while significantly enhancing the figure of merit (FOM) and resolution [17].

Collectively, these advances reflect the prevailing technological trend of integrating functional nanomaterials with structural optimization (e.g., waveguides and heterostructures), thereby overcoming traditional performance limits and laying a solid foundation for diverse practical applications.

### 2.2. Basic Principles of SPR

**Figure 1:**
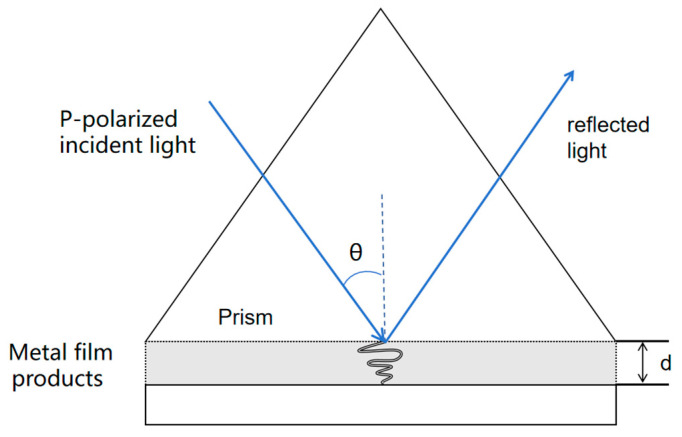
Principle of Surface Plasmon Resonance (SPR) sensing using the Kretschmann configuration. The incident light is focused through a high-refractive-index prism (below the metal film) to excite surface plasmon polaritons (SPPs) at the metal-sample interface. The sample (analyte) is in contact with the outer surface of the metal film.

When a polarized light beam irradiates the metal-film interface in a prism-coupled structure at an angle exceeding the critical angle, total internal reflection (TIR) occurs, generating an evanescent wave on the optical-dielectric side [1], as shown in Figure 1. By adjusting the incident angle, the wave vector of the evanescent wave can be matched with that of the surface plasmon wave on the metal surface. At this juncture, a substantial portion of the light energy is transferred to the surface plasmon mode, resulting in a drastic reduction in the intensity of reflected light and a resonant absorption peak [18]. The angle or wavelength corresponding to this peak is the resonance angle or resonance wavelength, respectively. Notably, resonance conditions are susceptible to minute changes in the refractive index of the medium adjacent to the metal surface. Since refractive index variations are proportional to the mass of biomolecules bound to the metal surface, SPR enables real-time, label-free detection of kinetic parameters in molecular interactions (e.g., binding rate constant k_a_, dissociation rate constant k^d^, and equilibrium dissociation constant K^D^) [1].

Despite its high sensitivity and real-time monitoring advantages, the traditional metal film structure of SPR sensors suffers from confined localized electromagnetic fields and insufficient sensitivity for low-abundance analytes—creating an urgent need for nanomaterial-based enhancement.

### 2.3. Signal Modulation of SPR Sensors

To adapt to diverse detection scenarios (e.g., precise laboratory analysis, rapid on-site testing), SPR sensors have evolved five distinct signal modulation methods—angular modulation, amplitude modulation, wavelength modulation, phase modulation, and Goos-Hänchen displacement modulation [18], each with unique sensitivity, stability, and structural complexity characteristics. Their performance and optimization strategies are detailed below.

#### 2.3.1. Angular Modulation

Angular modulation operates on the principle of fixing the wavelength of incident light and monitoring variations in the SPR spectrum by adjusting the incident angle; measurements are derived from the relationship curve between the resonance angle and the target variable. This method offers advantages such as simple operation, compact structure, and high sensitivity. It has reached a high level of maturity—serving as the mainstream modulation strategy for early commercial SPR sensors. In 2017, Zhou et al. designed an angularly modulated SPR spectrometer based on a five-layer Kretschmann structure (consisting of a prism, chromium adhesion layer, gold film, and sensing medium) [19]. By optimizing the thickness of the Cr/Au film via numerical simulation and introducing a reference beam to improve system stability, this approach achieved a sensitivity of 85 °/RIU and excellent repeatability with a standard deviation (SD) of 3.7 × 10^−6^ RIU. In 2022, Tong et al. designed a novel symmetric triangular metasurface-based SPR sensor. Through angular modulation, this structure achieved a maximum sensitivity of 364 °/RIU [20]. By optimizing the metal layer structure and dimensional parameters using COMSOL Multiphysics 5.1 software (combined with linear interpolation to enhance simulation accuracy), the sensor’s sensitivity was 1.7 times higher than that of conventional gold layer structures (135 °/RIU) [20].

The amplitude variation in single-angle signals remains limited for detecting low-concentration small molecules. Future optimization requires integrating nanomaterials to leverage their field-enhancement effects for further amplification.

#### 2.3.2. Amplitude Modulation

Amplitude modulation measures the impact of external variables on resonance by monitoring changes in light intensity at fixed incident angles and wavelengths [21]. Characterized by a simple structure and real-time response, this method is suitable for rapid detection scenarios [22,23]. However, it suffers from a narrow linear measurement range, susceptibility to ambient light interference, and poor stability in complex sample detection [4]. Zeng et al. designed an SPR imaging detection system to address these limitations, where two cameras capture 2D array reflection intensity data at different wavelengths [24]. Calculating the difference between the two datasets generates an image of refractive index changes. This dual-wavelength differential approach enables multi-channel high-throughput detection and enhances the refractive index resolution to 2.24 × 10^−6^ RIU, effectively mitigating environmental interference.

The amplitude modulation sensitivity still depends on the amplification of light intensity signals. Nanomaterials’ refractive index modulation capability can be further exploited to expand their linear range.

#### 2.3.3. Wavelength Modulation

Wavelength modulation uses a broadband light source with a fixed incident angle and monitors changes in the SPR wavelength [25,26]. This method is particularly well-suited for fiber-based SPR sensors (owing to their compact structure and easy integration). It offers high detection sensitivity and strong stability and has emerged as a key development direction for miniaturized SPR sensors in recent years [27].

In 2021, Chen et al. employed a long-wavelength light source to excite an SPR sensor [28], achieving a refractive index sensitivity of 11,773.93 nm/RIU and enabling the detection of target DNA at a concentration of 0.2 fmol/L. In 2024, Hu et al. extended the sensor’s operating wavelength to the near-infrared (NIR) region by using ZnO to adjust the sensor bandwidth [29]. They proposed a cascaded dual-channel fiber-optic SPR sensor, which achieved a maximum refractive index sensitivity of 9370.9 nm/RIU within the refractive index range of 1.333–1.415. A maximum temperature sensitivity of 7.97 nm/°C was obtained from 0 °C to 10 °C.

The core requirement of wavelength modulation is to enhance the “detectability” of resonant wavelength shifts. The LSPR effect of nanomaterials can amplify the signal amplitude of refractive index changes, facilitating the detection of ultra-low-concentration analytes [30].

#### 2.3.4. Phase Modulation

Phase-modulated SPR technology achieves high-sensitivity detection by measuring the phase difference between p-polarized and s-polarized components in reflected light (induced by changes in the surface refractive index) under fixed incident angles and wavelengths. Despite its exceptional sensitivity, this method suffers from a narrow dynamic range and susceptibility to phase jumps, hindering the full realization of its theoretical sensitivity [4]. To overcome this challenge, in 2020, Roman Kaňok et al. employed inverted fringe phase shift technology, enhancing the refractive index sensitivity to −226 rad/RIU and optimizing the detection limit to 4.4 × 10^−6^ RIU [31], thereby significantly improving measurement accuracy and reliability.

#### 2.3.5. Goos-Hänchen Displacement Modulation

When light undergoes total internal reflection (TIR) while propagating from a high-refractive-index medium to a low-refractive-index medium, the exit point of the reflected light does not coincide with the entry point of the incident light—resulting in a lateral displacement at the interface, known as the Goos-Hänchen (GH) shift. First detected by Goos and Hänchen in 1947 at the interface of two media with different refractive indices, the GH shift is typically tiny and challenging to measure directly [32].

A breakthrough was achieved in 2017; Li et al. developed a GH displacement-modulated SPR sensor based on a metasurface with nanogroove structures [33]. This sensor achieved a resolution of 10^−8^ RIU and could detect bovine serum albumin (BSA) at concentrations as low as 0.1 × 10^−8^ mol/L.

## 3. Nanomaterial-Enhanced SPR Sensors

The performance enhancement of surface plasmon resonance (SPR) sensors depends fundamentally on the optical properties of the nanomaterials employed, which determine both the excitation efficiency of the surface plasmon resonance and the quality of the resonant signal. To systematically analyze these materials, it is instructive to categorize them based on their Quality Factor (Q-factors), dielectric losses, and effective spectral regions [34]. Consequently, given their distinct roles in sustaining and modulating surface plasmons, nanomaterials are generally classified into three core types: Noble Metal Nanoparticles, Two-Dimensional (2D) Nanomaterials, and Magnetic Nanoparticles [4].

Noble Metal Nanoparticles, such as AuNPs and AgNPs, serve as the primary plasmonic hosts for supporting surface plasmons. Their performance is governed by the real and imaginary parts of their dielectric functions $\left(\epsilon (\omega )=\epsilon^{\prime }+i\epsilon^{\prime \prime }\right)$. While Silver (Ag) typically exhibits lower intrinsic losses, resulting in higher Q-factors and sharper resonances compared to Gold (Au), Au is often preferred for its superior chemical stability [35]. These nanoparticles utilize Localized Surface Plasmon Resonance (LSPR) to directly amplify the local electromagnetic field. In contrast, Two-Dimensional (2D) Nanomaterials, such as graphene and MXenes, primarily act as lossy modifiers or functional dielectric layers in the visible spectral region [36,37,38]. Although they significantly enhance sensitivity via high surface-to-volume ratios and superior adsorption efficiency, they often possess a substantial imaginary dielectric component. This introduces optical loss (damping), which can broaden the SPR curve and reduce the Q-factors if the layer thickness is not precisely optimized. However, in the Mid-Infrared (MIR) region, certain 2D materials distinguish themselves by being capable of supporting their own tunable plasmons.

Finally, Magnetic Nanoparticles (MNPs) function distinctly as enrichment agents [39,40]. Unlike the former two categories, they do not primarily serve as SPR exciters. Instead, they are utilized to suppress matrix interference through targeted magnetic separation and enrichment, thereby indirectly enhancing the signal-to-noise ratio in complex samples [39,41]. The following sections detail the specific mechanisms and applications of these material classes based on these physical distinctions.

The following sections detail the specific mechanisms and applications of these material classes based on these physical distinctions.

### 3.1. Noble Metal Nanoparticles

Noble metal nanoparticles (primarily AuNPs and AgNPs), also referred to as plasmonic nanoparticles, are characterized by their ability to sustain localized surface plasmon resonances (LSPR)—a collective oscillation of free electrons driven by incident light. This mechanism enables them to exhibit strong absorption in the visible and near-infrared (NIR) spectral regions and generate intense localized electric fields, making them essential for amplifying signals in SPR sensing applications. A key advantage is their ability to generate intense localized electric fields via the LSPR effect—critical for SPR sensors, as sensitivity is directly proportional to the intensity of the surface electric field (stronger fields enable more sensitive responses to refractive index changes) [42]. When the LSPR of noble metal nanoparticles couples with surface plasmon waves on the metal film of conventional SPR sensors, a “field enhancement effect” occurs, significantly amplifying the SPR signal. Different noble metal nanoparticles’ tunable biocompatibility and stability make them suitable for diverse applications (e.g., biomedical diagnostics, environmental monitoring) [43]. Among these, gold and silver nanoparticles are the most extensively studied and are discussed in detail below.

#### 3.1.1. Gold Nanoparticles (AuNPs)

AuNPs possess unique competitive advantages, including tunable optical properties, excellent biocompatibility, favorable surface modifiability, and precise size controllability [42]. With low biological toxicity and resistance to degradation in biological systems, AuNPs can be safely used for in vitro detection or in vivo targeted sensing. Their size can be precisely regulated (adapting to scenarios such as signal labeling and dark-field imaging), and their surfaces readily bind to recognition molecules (e.g., aptamers, antibodies), effectively enhancing detection specificity [44]. These properties make AuNPs the most widely used nanomaterial in bio-chemical SPR sensors. Several representative applications will be discussed in the following text:

##### Low-Molecular-Weight (LMW) Analyte Detection

Traditional SPR sensors face a universal challenge in detecting low-molecular-weight (LMW) analytes, whether they are environmental pollutants (e.g., heavy metal ions, phenolic compounds) or small organic molecules. Their low molecular weight induces negligible refractive index changes, often resulting in indistinguishable signals. However, AuNP-based mass amplification strategies have successfully bridged this gap. As highlighted in recent extensive reviews, these strategies are now effective for monitoring trace environmental hazards, such as mercury ions Hg^2+^ (using functionalized AuNPs) [45] and phenol (using Au-graphene quantum dots) [46], demonstrating the broad applicability of AuNP-enhanced sensing in complex matrices [44].

To address this, in 2016, Luo et al. proposed an AuNP-aptamer complex system where aptamers form stable complexes with unmodified AuNPs that are disrupted in the presence of target BPA molecules, leading to AuNP aggregation and a significant change in the solution’s refractive index [47]. When combined with fiber-optic SPR (FOSPR) probes, this method achieved a detection limit (LOD) of 0.0037 ng/mL for BPA—30 times more sensitive than traditional enzyme-linked immunosorbent assay (ELISA) methods—successfully resolving the challenge of small-molecule signal amplification.

Despite this versatility, limitations exist. These assays often rely on indirect competitive modes rather than direct binding, which increases experimental complexity [48,49]. Furthermore, the stability of AuNP aggregates is highly sensitive to ionic strength and pH. This poses a significant challenge when transferring methods from controlled buffers to complex real-world matrices—whether it be wastewater or biological fluids—where non-specific aggregation can lead to false positives [50].

##### Nucleic Acid Detection

In genetic diagnostics and virology, identifying low-abundance nucleic acids or viral markers with high specificity is a major challenge. AuNP-enhanced SPR sensors have demonstrated exceptional versatility in this domain. As detailed in recent reviews, specific applications have expanded beyond basic research to include the selective recognition of gene fragments (using thiolated DNA-modified AuNPs) [51] and the ultrasensitive detection of SARS-CoV-2 nucleocapsid proteins (utilizing large AuNPs for signal amplification) [52]. These advancements highlight the capability of AuNPs to facilitate the detection of infectious pathogens and genetic markers in complex biological fluids.

**Figure 2:**
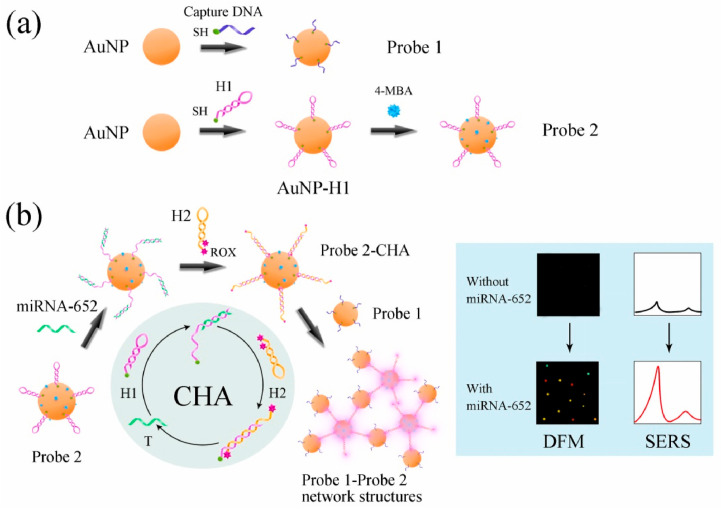
Schematic of the SPR/SERS dual-mode plasmonic biosensor based on CHA-induced AuNP networks for detecting miRNA-652. (**a**) Preparation of Probe 1 and Probe 2; (**b**) SPR/SERS dual-mode sensing strategy based on the CHA-induced AuNP network. DFM: dark-field microscopy; ROX: carboxy-X-rhodamine; 4-MBA: 4-mercaptobenzoic acid [53].

To improve accuracy, in 2021, Song et al. designed a dual-mode SPR/surface-enhanced Raman scattering (SERS) sensor [53]. By constructing a “capture DNA-fuel chain-signal probe” network using AuNPs, target miRNA-652 triggers a catalytic hairpin assembly (CHA) reaction. The LSPR of AuNPs simultaneously enhances the SPR optical signal (quantified via dark-field image integration of optical density) and amplifies the SERS molecular fingerprint signal (quantified by the ROX/4-mercaptobenzoic acid (4-MBA) ratio). For miRNA-652 in human serum, this sensor achieved an SPR LOD of 42.5 fM and a SERS LOD of 2.91 fM. Figure 2 illustrates this workflow, where Figure 2a details the preparation of functionalized AuNP probes, and Figure 2b depicts the specific CHA-induced assembly process that generates discernible dark-field microscopy (DFM) and SERS signals [53,54].

However, clinical translation faces technical hurdles. High-sensitivity assays often rely on complex amplification cascades (such as the CHA used above or enzyme-assisted strategies), which significantly increase assay time and reagent costs compared to direct detection. Additionally, the hybridization efficiency on solid surfaces is strictly governed by temperature and ionic strength; slight deviations in environmental conditions can lead to non-specific adsorption of the functionalized AuNPs, potentially generating background noise that compromises the limit of detection [50].

##### Biomacromolecule Detection

For larger biomacromolecules such as proteins and antibodies, the sensing challenge shifts from the mass limit to the preservation of biological activity and specificity. AuNPs have been widely utilized to construct biocompatible sensing interfaces that enhance signal intensity while maintaining receptor functionality. As summarized in recent reviews, applications in this domain have expanded to include the rapid diagnostic testing of viral infections (e.g., Dengue virus using gold nanospheres) [55] and the identification of fungal pathogens (e.g., Candida albicans antigen) [56]. In these applications, the LSPR effect of AuNPs serves to significantly amplify the refractive index shift induced by the binding of these large antigens.

In 2025, Wang et al. developed a photonic crystal fiber (PCF)-Au-polydopamine (PDA)-AuNP composite sensor to address this challenge [57]. This device enhances performance through a synergistic mechanism involving gold film, AuNPs, and PDA. Specifically, the gold film excites SPR, while AuNPs couple with the film via LSPR to amplify the local electric field. Furthermore, PDA magnifies refractive index signals and preserves protein activity through its excellent biocompatibility. Within the refractive index range of 1.335–1.365 (suitable for biological samples), the sensor achieved a refractive index sensitivity of 3523.10 nm/RIU—2.07 times higher than that of a standalone PCF-Au sensor. To detect rabbit immunoglobulin G (IgG), the PCF-Au-PDA-AuNP biosensor exhibited a biological sensitivity of 0.951 nm/(µg/mL) and an LOD of 0.021 µg/mL, effectively balancing high sensitivity and biocompatibility [57].

A critical limitation in this field is surface fouling. Large biomolecules tend to adsorb non-specifically onto metallic surfaces, leading to background noise that degrades the Limit of Detection (LOD) and reproducibility [50]. While surface modifications (like the PDA layer mentioned above) can mitigate this, ensuring the proper orientation of immobilized receptors on the curved surface of AuNPs remains a significant engineering challenge, as random orientation can obscure active binding sites and reduce sensor efficiency [58,59].

##### Circulating Tumor Cell (CTC) Detection

Cell detection represents the macroscopic limit of SPR sensing, where the focus is on capturing rare events in complex media. AuNP-functionalized interfaces have shown immense potential in this domain. As highlighted in recent reviews, applications have expanded beyond general cell detection to specific diagnosis of breast cancer and brain tumors [60,61]. Furthermore, these platforms are now utilized for the label-free, dynamic monitoring of cell evolutions, providing critical insights into cellular responses and drug efficacy in real-time [62].

**Figure 3:**
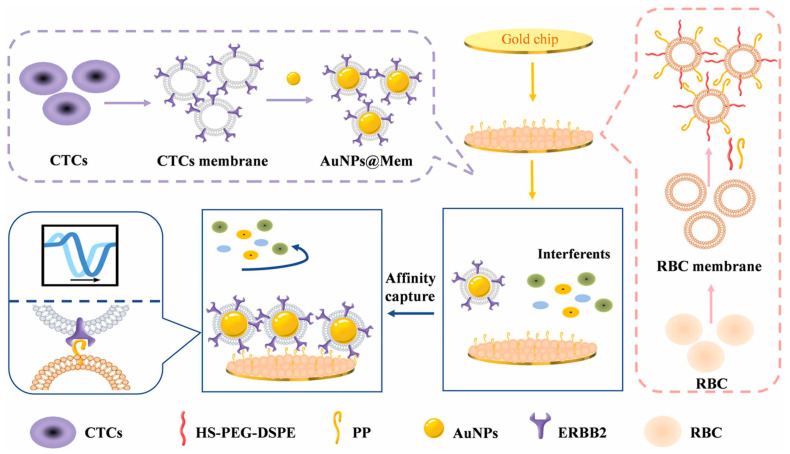
Schematic diagram of the biomimetic SPR sensing strategy for CTC detection [63]. The method proceeds in three steps: (i) Interface Construction: The gold chip is coated with a red blood cell membrane (RBCM) layer containing specific peptides to prevent non-specific adsorption of interferents; (ii) Target Capture: CTCs are specifically captured via the high-affinity binding between the surface peptides and ERBB2 proteins on the CTC membrane; (iii) Signal Amplification: AuNPs are employed to enhance the SPR response signal upon CTC binding.

To enable specific detection, in 2025, Liu et al. designed a red blood cell membrane (RBCM)-CTC functionalized AuNP system [63]. This approach achieves high-sensitivity detection through bionic recognition and signal amplification. First, RBCMs were coated onto the gold chip surface to reduce non-specific adsorption, while the ERBB2 protein on the CTC membrane surface specifically bound to peptides in the RBCMs. Finally, AuNPs functionalized on the CTC membrane acted as signal amplification agents to enhance the SPR response, as shown in Figure 3 [63]. This sensor exhibited a linear detection range for CTCs of 5 × 10^3^–1 × 10^6^ cells/mL, with an LOD as low as 868.11 cells/mL—successfully enabling the specific capture and detection of low-abundance CTCs in blood.

The primary limitation lies in the sample matrix interference. Whole blood is an extremely complex environment where high concentrations of non-target cells and proteins can physically block the sensor surface. As noted in the literature, non-specific binding remains a critical hurdle, as it directly affects the sensor’s selectivity and dynamic range. Although biomimetic coatings (like RBCMs) help, ensuring consistent capture efficiency across heterogeneous tumor cell populations without extensive sample pretreatment remains a significant challenge for clinical translation [25].

#### 3.1.2. Silver Nanoparticles (AgNPs)

Compared to AuNPs, AgNPs exhibit a more substantial LSPR effect (with a higher extinction coefficient in the visible light region) and higher catalytic activity—theoretically enabling more significant signal enhancement [42]. However, AgNPs suffer from poor stability (prone to oxidation and aggregation in solution) and weaker biocompatibility. In practical applications, composite structures (such as core–shell architectures or heterostructures) are required to improve their performance [4].

##### Environmental Small-Molecule Monitoring

**Figure 4:**
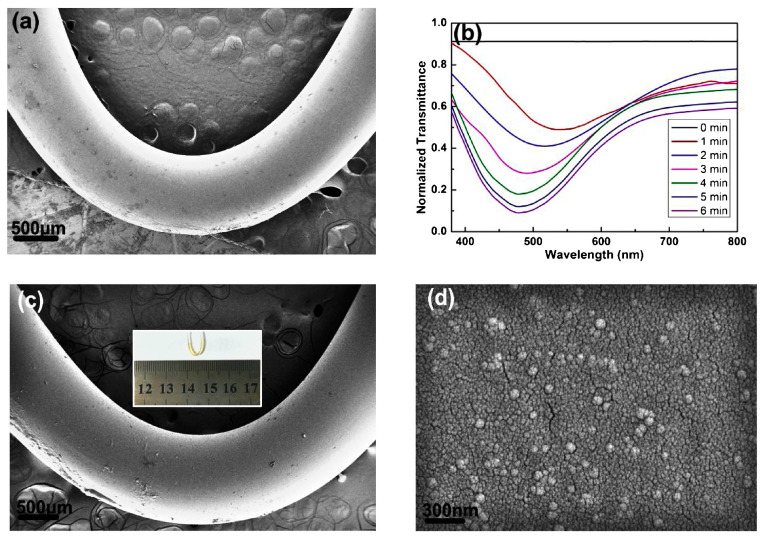
(**a**) Scanning electron microscope (SEM) image of a pure U-shaped bent fiber; (**b**) SPR spectra of U-shaped bent fiber probes at different laser-induced AgNP deposition times; (**c**) Optical image (inset in (**c**)); (**d**) SEM images of AgNPs deposited on the fiber surface [64].

For rapid detection of environmental small molecules (e.g., ethanol, glucose), in 2017, Zhang et al. designed a U-shaped bent fiber SPR sensor utilizing an optical fiber bent into a U-shape to enhance evanescent field interaction [64], optimizing its performance via a strategy that combines graphene protection with AgNP enhancement, as shown in Figure 4. Specifically: (a) the U-shaped bend increases the interaction between the SPR field and analytes; (b) the graphene layer prevents AgNP oxidation and aggregation, improving interface stability; (c) AgNPs amplify the SPR signal via the LSPR effect. By varying the laser exposure duration, the optimal AgNP deposition time was determined to be 4 min. Under these conditions, the sensor achieved SPR peak shifts of 32 nm and 16 nm when detecting a 90% ethanol aqueous solution (refractive index 1.3657) and a 20% glucose aqueous solution (refractive index 1.3557), respectively. It exhibited a sensitivity of 1198 nm/RIU, a response time of 3 s, and a recovery time of 80 s—meeting the requirements for rapid on-site detection.

##### Industrial Pollutant Detection

**Figure 5:**
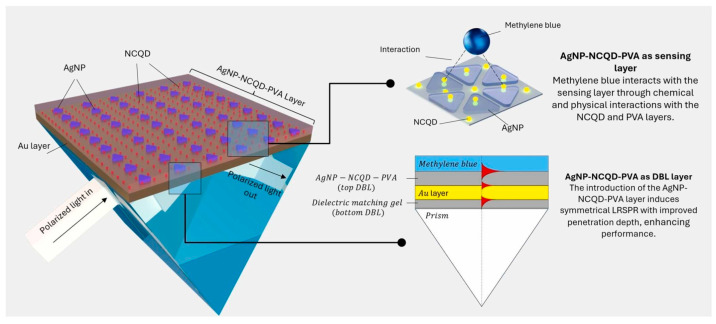
Schematic of the AgNP-NCQD-PVA composite sensor for methylene blue detection. NCQD: nitrogen-doped carbon quantum dot; PVA: polyvinyl alcohol [65].

Detecting industrial pollutants (e.g., methylene blue) requires balancing sensitivity and detection range. Traditional SPR methods are prone to signal saturation and exhibit a narrow linear range. To address this, in 2025, Bakar et al. developed an AgNP-nitrogen-doped carbon quantum dot (NCQD)-polyvinyl alcohol (PVA) composite system [65]. This composite serves as both the dielectric buffer layer and sensing material for long-range SPR (LRSPR) sensors Within this structure, NCQDs and PVA improve AgNP dispersion and stability. Furthermore, the LSPR of AgNPs couples with the long-range plasmonic waves of LRSPR, effectively amplifying the signal response. Experiments showed that for methylene blue, the sensor exhibited a slope of 4.2473 nm/ppm (R^2^ = 0.9231) in the 0.5–5 ppm concentration range and 0.5279 nm/ppm (R^2^ = 0.9629) in the 5–50 ppm range—significantly broader than that of a bare gold LRSPR sensor (limited to 0.5–5 ppm). Its FOM reached 0.0332 ppm^−1^, with an affinity of 1.642 × 10^4^ M^−1^—outperforming the bare gold sensor and making it suitable for wide-range pollutant detection, as illustrated in Figure 5 [65].

##### Biological Detection

**Figure 6:**
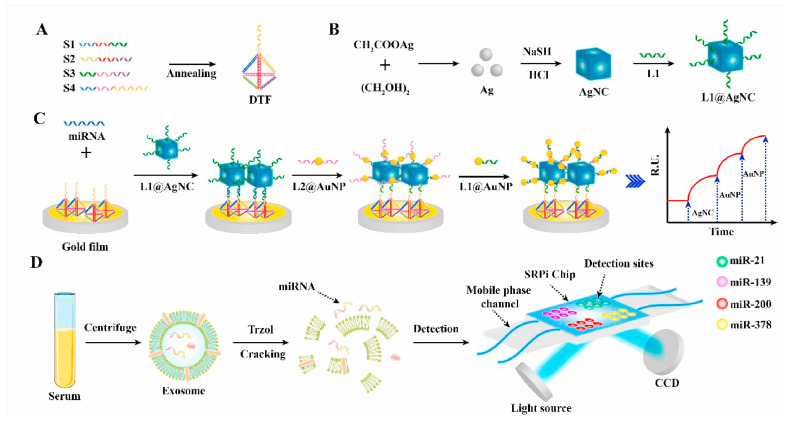
Schematic of the SPRi-based biosensor for detecting multiple exosomal miRNAs. (**A**) Self-assembly of DNA tetrahedral frameworks (DTFs); (**B**) Synthesis of single-stranded DNA-functionalized silver nanocubes (AgNCs); (**C**) Sensing mechanism for miRNA capture and signal amplification; (**D**) SPRi chip design for multi-channel detection. SPRi: surface plasmon resonance imaging [66].

To overcome the poor biocompatibility of AgNPs, researchers developed a “gold-on-silver” (Au-on-Ag) heterostructure—preserving the strong LSPR effect of AgNPs while leveraging the biocompatibility of gold to shield AgNPs and reduce toxicity. A notable application, Wu et al. integrated this heterostructure with a DNA tetrahedral framework (DTF) to construct an SPR imaging (SPRi) biosensor in 2021 [66]. The DTF specifically captures miRNAs from non-small cell lung cancer (NSCLC) exosomes, while the Au-on-Ag heterostructure amplifies the SPR signal. This sensor exhibited a detection range of 2 fM–20 nM and an LOD of 1.68 fM, enabling the simultaneous detection of multiple exosomal miRNAs to support early cancer diagnosis, as detailed in Figure 6.

#### 3.1.3. Advanced 1D and 2D Plasmonic Nanostructures

While spherical nanoparticles (0D) are widely used, recent advancements have shifted toward anisotropic one-dimensional (1D) and two-dimensional (2D) nanostructures to overcome the limitations of single-mode resonance and limited field confinement [67,68,69]. 1D nanostructures, such as gold nanorods (AuNRs) and nanowires, exhibit two distinct LSPR modes: a transverse mode and a longitudinal mode [54,70]. The longitudinal mode is highly tunable by adjusting the aspect ratio, allowing the resonance wavelength to be precisely shifted from the visible to the near-infrared (NIR) region (650–1350 nm). This tunability is particularly advantageous for analyzing biological samples (e.g., blood, tissue), as the NIR window minimizes photodamage and autofluorescence interference [70,71,72].

Furthermore, 2D nanostructures like nanotriangles, nanostars, and nanoplates offer superior field enhancement capabilities due to the lightning rod effect. The sharp tips and edges of these structures serve as electromagnetic hot spots [73], where the local electric field intensity is amplified by orders of magnitude compared to smooth spherical surfaces [54]. For instance, recent studies have introduced ordered arrays of 2D nanodisks and nanoholes, which not only enhance the near-field intensity but also suppress radiative damping, leading to narrower spectral linewidths and higher Figures of Merit (FOM). These advanced morphologies represent a critical evolution in SPR sensor design, transitioning from simple signal amplification to tunable, high-precision sensing platforms [74,75,76,77,78,79,80].

### 3.2. Magnetic Nanoparticles (MNPs)

Unlike the “signal amplification” mechanism of noble metal nanoparticles, the core advantage of MNPs (e.g., Fe_3_O_4_, CoFe_2_O_4_) lies in their dual capability for targeted enrichment and interface optimization.

- Targeted enrichment: Using external magnetic fields, MNPs enable rapid separation and enrichment of target analytes from complex matrices (e.g., serum, food extracts, soil solutions), reducing interference from non-specific adsorption [42].
- Interface optimization: MNP surfaces can be easily modified with recognition molecules (e.g., antibodies, enzymes, nucleic acids), enhancing target binding stability.

By designing core–shell structures (e.g., Fe_3_O_4_@Au, Fe_3_O_4_@PDA), the chemical stability and biocompatibility of MNPs can be further improved—directly addressing the bottlenecks of traditional SPR (substantial interference from complex matrices, difficulty in detecting low-concentration analytes). Representative applications are as follows.

#### 3.2.1. Food Safety Testing

Ochratoxin A (OTA), a common mycotoxin in grains and nuts, requires complex sample pretreatment in traditional SPR detection to avoid interference from food matrices. Lucian-Gabriel et al. developed a label-free MNP-based OTA immunosensor to simplify pretreatment in 2011 [81]. A gold electrode was modified with a bovine serum albumin (BSA) conjugate to form an anti-adsorption coating, followed by conjugation of OTA antibodies to MNPs. A strong magnetic field immobilized the MNP-antibody complex onto the electrode surface; the magnetic properties of MNPs enabled rapid OTA enrichment, while antibodies ensured specific binding. This sensor exhibited a stable response in the 0.01–5 ng/mL range, with an LOD of 0.94 ng/mL—enabling direct detection of food samples without complex pretreatment [81].

#### 3.2.2. Pesticide Residue Testing

In agricultural products, pesticide residues (e.g., thiabendazole (TBZ)) often exist at the ng/mL level, where traditional SPR signals are easily masked by environmental noise.

To enhance detection sensitivity, Li et al. designed Fe_3_O_4_@Au@PDA core–shell MNPs in 2025, achieving performance enhancement via a “triple mechanism”: (a) the Fe_3_O_4_ core enriches TBZ from soil extracts and fruit/vegetable juices using a magnetic field; (b) the Au shell couples with SPPs on the SPR metal film to amplify the local electric field; (c) the PDA layer acts as a reducing agent to promote Au nanoparticle deposition and enhances monoclonal antibody (Ab) immobilization via amino and hydroxyl groups [82]. The optimized sensor achieved a TBZ LOD of 0.61 ng/mL and a linear range of 1–200 ng/mL—meeting the requirements for trace pesticide detection in agricultural products.

#### 3.2.3. Bioenzyme Sensing Bioenzyme

**Figure 7:**
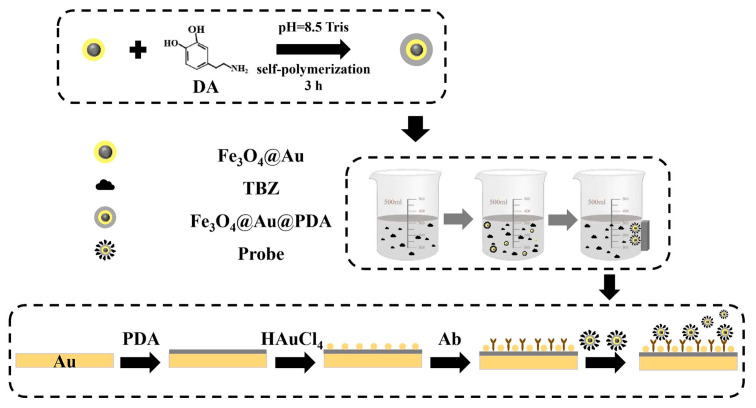
Schematic of the fabrication process for the Fe_3_O_4_@Au@PDA core–shell MNP sensor for TBZ detection. TBZ: thiabendazole; PDA: polydopamine [82].

The activity of biological enzymes (e.g., lactose dehydrogenase (LDH)) is easily affected by environmental factors. Traditional SPR sensors often cause enzyme denaturation during immobilization, compromising detection stability. A solution for stable enzyme sensing, Hassan et al. developed a graphitic phase carbon nitride/magnetic chitosan (g-C_3_N_4_/MNP/CS) composite in 2024 [83]. The amine groups of CS bond to gold monolayers, g-C_3_N_4_ provides a high specific surface area, and MNPs magnetically regulate the immobilization position and density of LDH, as illustrated in Figure 7. This tripartite synergy protects LDH activity while enhancing transient field intensity. The sensor achieved a sensitivity of 238 °/RIU, an LOD of 5 μM, and a linear range of 0.01–100 mM—exhibiting significantly superior enzyme reusability and stability compared to conventional sensors.

### 3.3. Two-Dimensional (2D) Nanomaterials

2D nanomaterials (e.g., graphene, MoS_2_, black phosphorus, MXenes) [42] possess core characteristics of atomic-scale thickness, ultra-large specific surface area, and excellent optoelectronic properties, which systematically enhance SPR sensor performance in four dimensions. First, the large specific surface area and abundant functional groups (e.g., hydroxyl, amino, carboxyl) provide numerous active sites for efficient analyte binding, thereby enhancing target capture efficiency [36,37,38]. Second, strong π–π stacking interactions with aromatic biomolecules (e.g., proteins, nucleic acids) improve detection specificity by reducing non-specific adsorption [84,85,86]. Additionally, these materials enhance stability by coating metal films or nanoparticles to prevent oxidation (e.g., graphene protecting AgNPs) [87]. Finally, the work function difference between 2D nanomaterials and metal films regulates interfacial electric field strength, accelerating charge transfer to further intensify electric fields [37].

Based on compositional differences, 2D nanomaterials can be divided into graphene-based materials and non-carbon 2D nanomaterials, each with distinct application focuses.

#### 3.3.1. Graphene-Based Nanomaterials

Graphene exhibits excellent optical (high light transmittance), electrical (high carrier mobility), and mechanical properties [42], along with superior biocompatibility—making it the preferred 2D material for SPR enhancement in biomedical applications. The core of its enhancement mechanism lies in chemical potential modulation; the chemical potential of graphene can be adjusted via external electric fields or layer thickness [4]. When the chemical potential reaches a critical threshold, the sensitivity of SPR sensors increases significantly due to an increase in the real part (or optical conductivity) of the dielectric constant [88]. This results in a larger angle shift in attenuated total reflection (ATR) spectra, providing a precise means for performance tuning. The representative applications are Mid-Infrared (MIR) Band Biological Detection [88], Heavy Metal Ion Detection [89], and Glucose Detection [87], and they will be discussed in more detail in the following sections:

##### Mid-Infrared (MIR) Band Biological Detection

**Figure 8:**
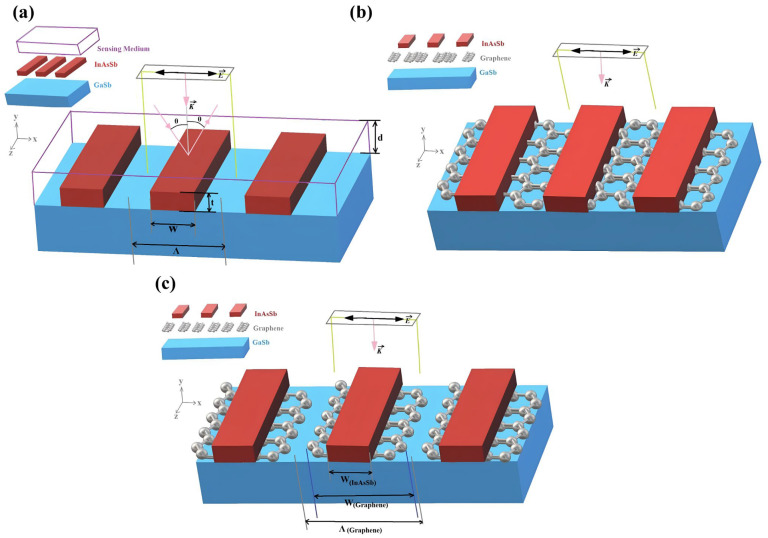
Schematic configuration of the mid-infrared (MIR) SPR sensor based on a graphene-InAsSb nanoribbon composite. (**a**) The basic sensor structure utilizing periodic arrays of highly doped InAsSb semiconductor nanoribbons to excite surface plasmons; (**b**) The enhanced composite structure incorporating graphene layers integrated with the semiconductor nanoribbons to modulate the chemical potential and enhance field confinement; (**c**) A detailed cross-sectional view of the sensing interface, illustrating the geometric alignment of graphene with the InAsSb nanoribbons and the interaction with incident light [90].

Traditional SPR sensors primarily operate in the visible light spectrum, making it challenging to utilize the infrared “fingerprint” vibrational information of molecules, resulting in insufficient specificity [4]. Miandoab et al. designed a highly doped semiconductor (InAsSb) nanoribbon-graphene composite sensor to enable MIR-based specific detection in 2022 [90]. Nanoribbons are quasi-one-dimensional nanostructures with high aspect ratios, typically synthesized via lithographic patterning to form periodic arrays that effectively confine light at the nanoscale. In this specific application, InAsSb nanoribbons provide the fundamental SPR effect, while graphene nanoribbons enhance MIR localized electric fields via chemical potential modulation and achieve “vibrational fingerprint” detection by resonating with MIR radiation and molecular vibrations. After incorporating graphene, the sensor’s sensitivity increased from 918 nm/RIU to 2315 nm/RIU [72]. By adjusting the chemical potential of graphene, the sensor can be adapted to detect different biomolecules, as shown in Figure 8.

##### Heavy Metal Ion Detection

**Figure 9:**
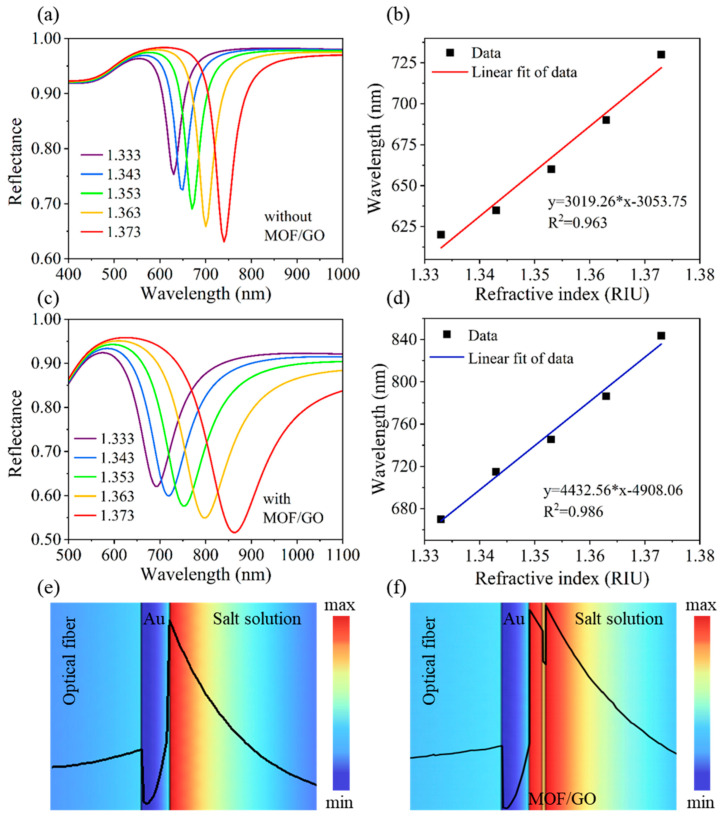
Reflectance spectra of different refractive index for the (**a**) MOF/GO-uncoated and (**c**) MOF/GO-coated optical fiber SPR sensors. Linear fit of resonance wavelength of different refractive index for the (**b**) MOF/GO-uncoated and (**d**) MOF/GO-coated optical fiber SPR sensors. Electric field profile for the (**e**) MOF/GO-uncoated and (**f**) MOF/GO-coated optical fiber SPR sensors, respectively [92].

Detecting environmental heavy metal ions (e.g., Pb^2+^) at the pM level often requires recognition elements such as DNAenzymes in traditional SPR sensors [89,91]. To improve sensitivity and selectivity, Li et al. developed a gold-based fiber SPR sensor in 2025, enhancing its performance by modifying the sensing interface with a metal–organic framework (MOF)/graphene oxide (GO) composite layer [92]. Within this composite, the GO component provides a high specific surface area to enhance adsorption, while the MOF improves selectivity via the size-sieving effect. DNA enzymes immobilized on the composite layer are activated upon binding to Pb^2+^, cleaving the substrate chain and detaching coupled gold nanoparticles—triggering a refractive index decrease and an SPR spectral blue shift. This sensor achieved a refractive index sensitivity of 4248.64 nm/RIU and a Pb^2+^ LOD of 3.419 pM, with excellent selectivity against other heavy metal ions, as shown in Figure 9 [92].

##### Glucose Detection

**Figure 10:**
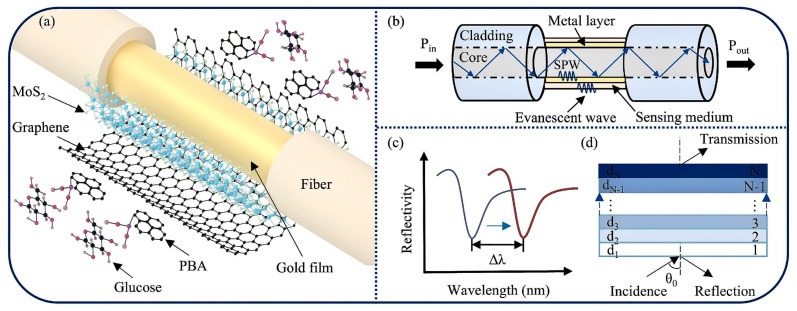
Schematic representation of the fiber-optic SPR (FO-SPR) sensor for glucose detection. (**a**) illustrates the overall sensor design where light propagates through the fiber core. (**b**) details the sensing principle, showing the interaction between the evanescent wave and the sensing medium at the metal-coated region. (**c**) displays the reflectance spectra, where the resonance wavelength shift (Δλ) indicates changes in the refractive index. (**d**) depicts the multilayer sensing interface configuration, consisting of the metal film, MoS2 layers, graphene layers, and the target analyte [93].

Blood glucose detection requires high sensitivity and anti-interference capability to effectively avoid interference from other sugars in the blood. Ma et al. present a high-performance solution. They developed a fiber-optic SPR (FO-SPR) sensor in 2025, optimized via a MoS_2_–graphene heterostructure functionalized with 1-pyrene boronic acid (PBA),where the high refractive index of MoS_2_ enhances SPR signals, graphene improves interface stability and electron transfer efficiency, and PBA specifically binds to glucose hydroxyl groups [93]. Sensors modified with two layers of MoS_2_ and two layers of graphene achieved a sensitivity of 8631.54 nm/RIU, further increasing to 12,593.06 nm/RIU after PBA modification. They exhibited excellent linearity in the 0–500 mg/dL range (covering the normal human blood glucose range), making them suitable for real-time glucose monitoring, as depicted in Figure 10 [93].

#### 3.3.2. Non-Carbon 2D Nanomaterials

Graphene exhibits low optical absorption, limiting its ability to promote charge transfer with metal films [42]. In contrast, non-carbon 2D nanomaterials (e.g., WS_2_, MoS_2_, MXenes, indium tin oxide (ITO)) possess higher optical absorption and refractive indices. Some materials (e.g., MXenes) also feature abundant surface functional groups, offering unique advantages in environmental monitoring and food testing applications [94]. Representative studies are as follows.

##### Food Component Detection

**Figure 11:**
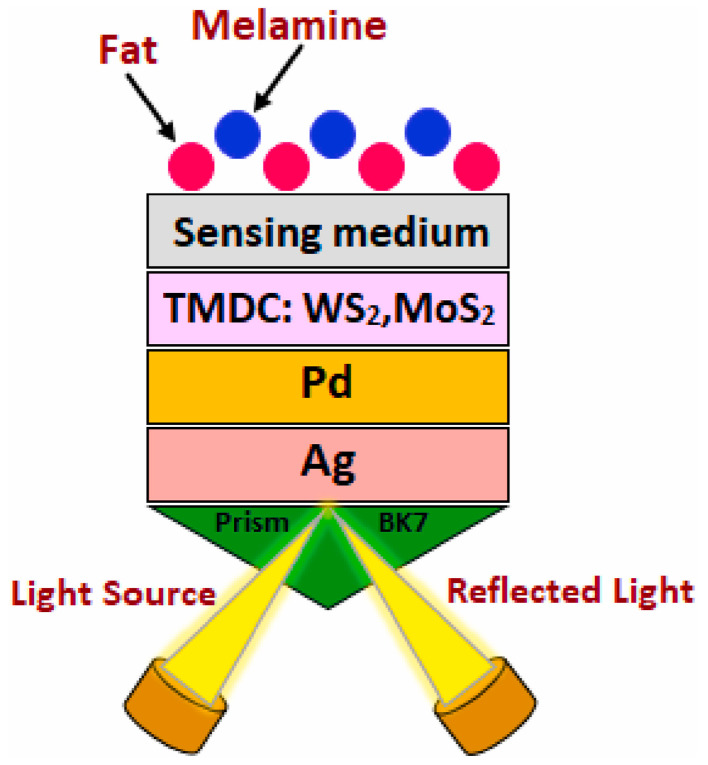
Schematic of the TMDC-based SPR sensor for detecting fat and melamine concentrations in milk. TMDC: transition metal dichalcogenide (WS_2_, MoS_2_); BK7: a common borosilicate crown glass used as the prism material [96].

Simultaneous detection of fat and melamine in milk requires sensors with multi-component response capabilities; traditional SPR methods often suffer from signal overlap [95]. To achieve simultaneous detection, Leila et al. designed a WS_2_/MoS_2_-silver-palladium (Ag-Pd) bimetallic layer SPR sensor in 2024 [96]. WS_2_ exhibits strong adsorption for fats, MoS_2_ (a transition metal dichalcogenide, TMDC) shows high specificity for melamine adsorption [97], the Ag layer provides the fundamental SPR effect, and the Pd layer enhances stability while synergistically amplifying the electric field with TMDCs.as shown in Figure 11. This sensor achieved a sensitivity of 409.36 °/RIU and an LOD of 0.0085 RIU for fat detection, and a sensitivity of 248.96 °/RIU and an LOD of 0.0140 RIU for melamine detection—enabling simultaneous quantitative detection of both components without interference from other milk constituents.

##### Mid-Infrared (MIR) SPR Fiber Optic Sensing

**Figure 12:**
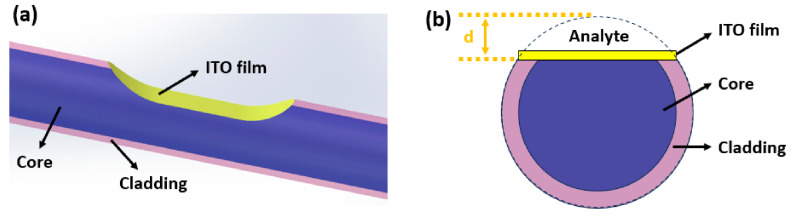
Schematic of the D-shaped ITO-coated MIR SPR fiber sensor. (**a**) Overall sensor structure; (**b**) Cross-section of the sensor (polished depth d is indicated). ITO: indium tin oxide; MIR: mid-infrared [98].

Traditional visible-light SPR sensors exhibit rapid evanescent wave attenuation (detection depth ~1/10 of the wavelength), making detecting thick-layer samples or large-volume analytes challenging. In contrast, MIR evanescent waves decay more slowly (detection depth ~1/3 of the wavelength), significantly enhancing detection depth. Lin et al. in 2025, proposed a MIR SPR fiber sensor using multimode quartz fibers processed into a D-shaped geometry [98]. The utilization of multimode quartz ensures mechanical robustness and efficient light coupling, while the unique D-shaped profile exposes the evanescent field to the sensing interface. This structure is coated with a 105 nm ITO layer to exploit MIR advantages [39]. ITO, a transparent conductive material, exhibits excellent plasmonic properties in the MIR band, enabling SPR excitation; the D-shaped structure increases the fiber’s contact area with analytes. Experimental results showed resonance near 2700 nm, with a sensitivity of 1065.70 nm/RIU across the refractive index range of 1.33–1.42. Furthermore, alignment with the MIR molecular fingerprint region enables gas detection (e.g., volatile organic compounds (VOCs)), expanding the application scope of SPR sensors, as shown in Figure 12.

### 3.4. Comparative Analysis of Nanomaterial Enhancement Strategies

While each nanomaterial category discussed above offers unique enhancement pathways—ranging from LSPR-mediated signal amplification in noble metals to interface optimization provided by 2D materials—practical sensor design requires balancing these benefits against inherent limitations. For instance, while AgNPs and anisotropic nanostructures offer superior field confinement, they often compromise chemical stability compared to inert AuNPs. Similarly, 2D materials like graphene provide excellent surface area but require hybridization with metals to excite plasmons effectively. To facilitate the selection of appropriate materials for specific sensing scenarios, Table 1 presents a comparative benchmarking of these nanomaterial classes, highlighting their respective enhancement mechanisms, stability profiles, and critical trade-offs.

## 4. Advances in Nanomaterial-Integrated SPR Sensors with Multi-Technology Convergence

### 4.1. Nanomaterials and Intelligent Algorithms

Traditional nanocomposite structures (e.g., multilayer films, heterogeneous nanoparticles) rely on simulation methods such as the Finite Difference Time Domain (FDTD) and Rigorous Coupled-Wave Analysis (RCWA). These methods require several hours to days of computation and struggle to cover extensive design spaces (e.g., simultaneous optimization of five or more structural parameters) [99]. In contrast, intelligent algorithms (e.g., machine learning (ML), explainable AI (XAI)) replace empirical trial-and-error with “data-driven modeling,” significantly improving nanostructure design efficiency while ensuring the physical feasibility of optimization results [100,101].

**Figure 13:**
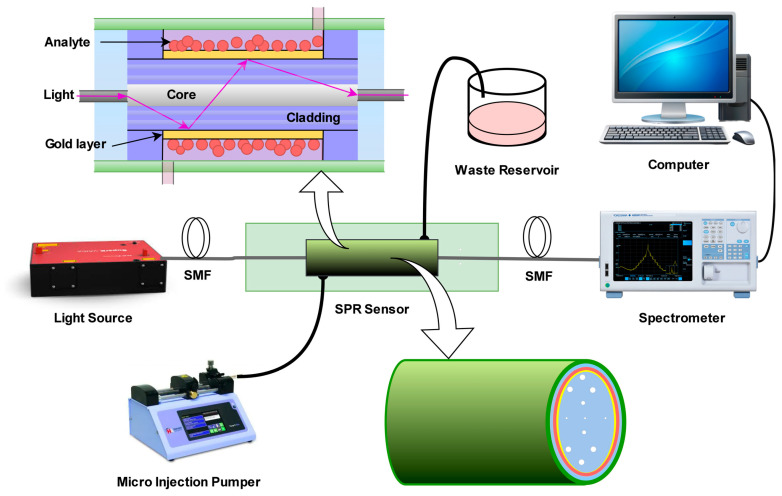
Schematic of the PCF-SPR biosensor designed by Mst. Rokeya Khatun et al. PCF: photonic crystal fiber; SMF: single-mode fiber [17].

**Figure 14:**
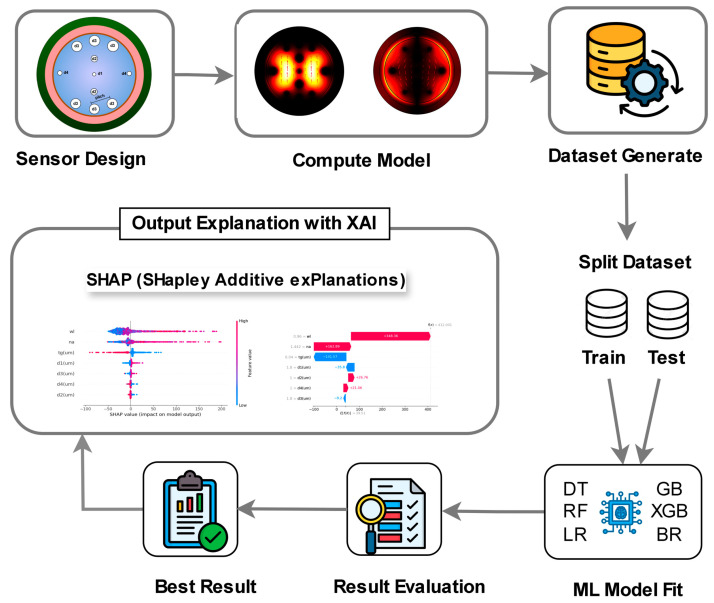
Schematic of the algorithm combining XAI and ML for PCF-SPR sensor optimization. XAI: explainable AI; ML: machine learning; SHAP: Shapley Additive exPlanations; GB: Gradient Boosting; XGB: Extreme Gradient Boosting [17].

In 2025, Khatun et al. designed a PCF-SPR sensor by integrating nanomaterials with intelligent algorithms, utilizing the sensor structure illustrated in Figure 13 [17]. First, extensive datasets of structural parameters (gold layer thickness, pore spacing, wavelength) vs. performance metrics (sensitivity, FOM) were generated. ML models (e.g., Gradient Boosting (GB), Extreme Gradient Boosting (XGB)) were then trained to achieve millisecond-level performance prediction (compared to hours for traditional FDTD methods) [4]. Subsequently, the Shapley Additive exPlanations (SHAP) method was used to quantify parameter contributions, identifying wavelength (SHAP value: +412.001), analyte refractive index (+348.36), and gold layer thickness (−141.57) as core factors influencing sensitivity—consistent with SPP propagation principles (gold layer thickness affects plasmonic wave decay length), thus avoiding the black box issue of ML models [101]. This comprehensive algorithmic framework is schematically represented in Figure 14. The optimized PCF-SPR sensor achieved maximum wavelength sensitivity beyond conventional limits, with significantly enhanced FOM and resolution—establishing a paradigm for efficient nanocomposite structure design [17].

### 4.2. Nanomaterials and Multimodal Detection

SPR excels in real-time, label-free monitoring of molecular interaction kinetics but lacks chemical specificity as it relies solely on refractive index changes [4]. In contrast, SERS enables molecular fingerprinting by distinguishing molecules via Raman peak positions and ultra-high sensitivity but is often limited in monitoring fast dynamic processes. Integrating SPR and SERS enables synergistic real-time monitoring and specific recognition, with nanomaterials as the core carrier for dual-signal enhancement [102].

**Figure 15:**
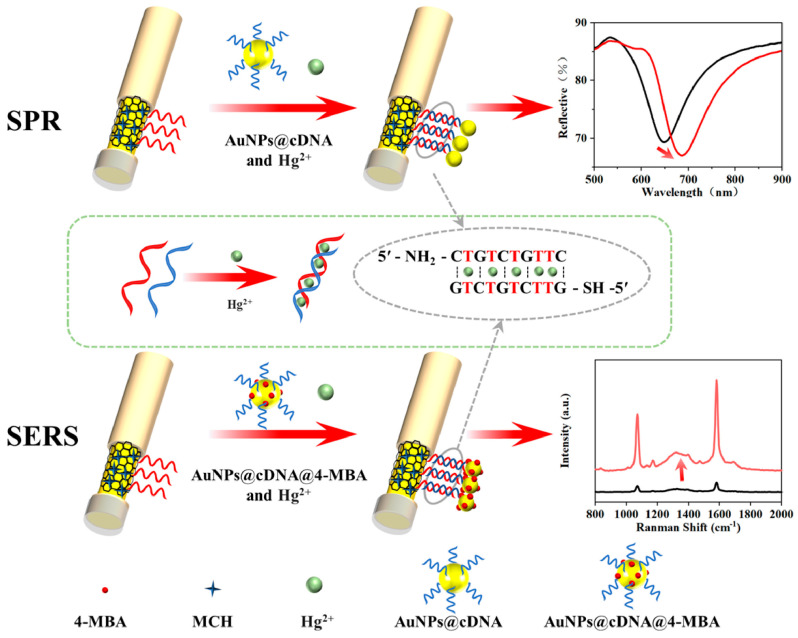
Schematic of the dual-mode SPR/SERS fiber optic sensor designed by Li et al. [103].

Li et al. proposed a cutting-edge application in 2025, developing a dual-mode fiber sensor that capitalizes on the synergistic coupling between SPR and SERS to overcome individual limitations [103]. This synergy is realized through AuNPs, which function as dual-purpose enhancement units: in the SPR mode, AuNP-based LSPR amplifies the resonant wavelength shift signals to enable real-time dynamic analysis, while in the SERS mode, the nanoparticles provide electromagnetic (EM) enhancement combined with charge transfer (CT) enhancement from an Au/graphene oxide (GO) layer. This dual mechanism reduces the detection limit from the nanomolar (nM) range (typical of SPR alone) to the femtomolar (fM) range. Significantly, in cases of non-specific adsorption (e.g., serum impurities) where SPR signals shift but SERS shows no characteristic peaks, the sensor utilizes the disparity between the observed SPR spectral shift and the absence of SERS characteristic peaks to effectively eliminate false positives [103]. This integration expands the dynamic range to the micromolar (μM) level, covering the concentration range of most clinical biomarkers, as illustrated in Figure 15.

### 4.3. Nanomaterials and Microfluidics: Enabling Point-of-Care (POC) Diagnostics

Point-of-Care (POC) diagnostics fundamentally require portability, automation, and speed; however, traditional SPR sensors are typically bulky and necessitate complex sample pretreatment, rendering them unsuitable for primary care hospitals or field testing [4]. Microfluidic technology enables integrated sample preparation-detection-result output via precise control of microliter-scale fluids; incorporating nanomaterials further enhances detection performance, driving the decentralized application of SPR technology.

In 2023, Liu et al. developed a lateral flow strip based POC molecular diagnostics platform [104]. This microfluidic cassette integrates four modules: sample lysis, nucleic acid extraction, isothermal amplification, and SPR detection. Saliva samples undergo automated processing via microchannels, and AuNP-modified nucleic acid probes amplify SPR signals via the LSPR effect [4,104]. The platform completes the entire process from sample to result within 15 min, achieving 98% accuracy in detecting SARS-CoV-2 nucleic acids which is comparable to quantitative polymerase chain reaction (qPCR). With a volume only one-tenth that of traditional SPR instruments and supporting handheld operation, this integrated microfluidic lateral flow system provides a viable solution for rapid diagnosis in remote areas and disaster sites [104].

To provide a comprehensive overview of how these integrated strategies translate into tangible performance gains, Table 2 summarizes the key research directions, core technologies, and representative quantitative breakthroughs achieved in recent studies. This compilation highlights the specific advantages of each platform in addressing critical challenges such as sensitivity limits, detection speed, and result reliability.

## 5. Challenges and Future Outlook

### 5.1. Challenges

Despite significant progress, nanomaterial-enhanced SPR sensors face critical bottlenecks that hinder their transition from laboratory prototypes to robust clinical devices. Beyond the issues of cost and manufacturing, the stability of real-time performance under complex environmental conditions remains a primary challenge [4].

- Environmental Cross-Sensitivity and Thermal Stability: SPR sensors are inherently sensitive to temperature fluctuations, which alter both the refractive index of the bulk buffer (dn/dT) and the electron collision frequency in the metal film [48]. A fluctuation of just 1 °C can induce a baseline drift comparable to the signal of low-abundance analytes, leading to false positives [105]. Most nanomaterial-based designs currently lack integrated temperature compensation mechanisms (such as dual-channel self-referencing or thermo-optic compensating materials like ZnO), limiting their reliability in uncontrolled field environments.
- Chemical Stability and Oxidation: For non-gold plasmonic materials (e.g., AgNPs, CuNPs, and MXenes), chemical instability is a major barrier. As noted in recent studies, silver and copper nanoparticles are prone to rapid oxidation in aqueous physiological buffers [58]. This oxidation dampens the LSPR peak intensity and causes a blue shift in the resonance wavelength, degrading sensor accuracy within minutes of operation. While core–shell strategies (e.g., Ag@SiO_2_ Ag@Au) offer partial mitigation, they often increase the distance between the analyte and the evanescent field, creating a trade-off between stability and sensitivity [4].
- Reproducibility and Standardization: Laboratory-fabricated nanocomposites often exhibit batch-to-batch variations in morphology and surface coverage. This inconsistency makes it difficult to establish a universal calibration curve, which is fatal for quantitative clinical diagnostics. Furthermore, the lack of standardized interfaces compatible with medical Laboratory Information Systems (LIS) delays regulatory approval [4].

### 5.2. Future Outlook

To address these challenges, future research must achieve breakthroughs in technical details to advance SPR sensors toward clinical practicality and industrialization. Key directions include:

- Novel Nanomaterial Development: The MIR compatibility of MXenes such as Ti_3_C_2_T_x_ stems from their tunable dielectric constant [106]. By adjusting the content of surface -OH and -F functional groups, the real part of the dielectric constant can be tuned to the 10–20 range, matching the excitation requirements of MIR SPPs and overcoming the application limitations of traditional noble metals in the MIR band [94].
- Low-Cost Alternative Exploration: Low-cost copper nanoparticles (CuNPs) can be synthesized using simple methods such as electrodeposition or green synthesis with ascorbic acid [107,108]. Compared to gold nanoparticles (AuNPs),copper nanoparticles (CuNPs) exhibit a significantly more economical real part of the dielectric constant, and coating them with silica (SiO_2_) effectively enhances their stability [45,109,110].
- Flexible Wearable SPR Sensor Development: Sensors require integrated solutions for continuous power supply such as Near Field Communication and piezoelectric nanogenerators [111,112], as well as long-term stability achieved through protective coatings to function reliably in complex physiological environments like sweat [27,112,113,114].
- AI-Driven Nano-Enhanced SPR Sensing Networks: The collaborative architecture of “edge nodes + cloud AI” enables efficient data processing and intelligent analysis. At the edge, deployed devices such as LSPR biosensors collect physiological data like sweat and cortisol in real time [115]. Implementing a collaborative architecture of edge nodes and cloud AI enables efficient data processing, dynamic early warning systems, and global model optimization [100,101].
- Global Safety Regulation and Standardization: The global regulatory environment is actively addressing biosafety demands, yet establishing unified standards remains a critical challenge. The primary obstacle to uniform standardization lies in the physicochemical diversity of nanomaterials, which often interferes with traditional toxicity assays, leading to inconsistent results. Future advancements must focus on international harmonization to develop robust test methods, shifting the industry toward a mandatory and systematic global regulatory framework [116].

## 6. Conclusions

This review systematically examines the evolution of SPR sensors from traditional thin-film configurations to advanced nanomaterial-enhanced platforms. While conventional SPR sensors have established a foundation for label-free detection through various modulation strategies, they remain constrained by limited electromagnetic field confinement and insufficient sensitivity for low-abundance analytes. Nanomaterial integration has emerged as the critical solution to bridge this gap.

Our analysis of three core nanomaterial categories highlights their distinct yet complementary roles: Noble metal nanoparticles (including spherical 0D and anisotropic 1D/2D structures) drive signal amplification via LSPR and electromagnetic hot spots; Magnetic nanoparticles address the challenge of matrix interference through targeted enrichment and separation; and 2D nanomaterials optimize the sensing interface by enhancing stability and providing tunable optoelectronic properties. However, as individual materials face inherent constraints—such as the oxidative instability of AgNPs or the biocompatibility issues of MNPs—the field is shifting towards synergistic composite designs to balance sensitivity with practical applicability.

Furthermore, the convergence of these nanomaterials with emerging technologies has accelerated the transition from laboratory research to clinical practice. The integration of intelligent algorithms (ML/AI) has revolutionized nanostructure design efficiency; multimodal detection (e.g., SPR/SERS) has significantly improved detection reliability by eliminating false positives; and microfluidic systems have enabled portable, automated Point-of-Care (POC) diagnostics.

Despite these strides, industrialization faces substantial bottlenecks, including the environmental instability of nanomaterials (thermal and oxidative degradation), poor batch-to-batch reproducibility, lack of standardization, and gaps in biosafety assessment. Future research must prioritize the development of robust, environmentally stable novel materials (e.g., MXenes, oxidation-resistant 1D/2D structures), low-cost manufacturing techniques, and integrated wearable systems. Addressing these challenges is essential to transforming nanomaterial-enhanced SPR sensors into standardized, reliable tools for next-generation biomedical and environmental monitoring.

## Figures and Tables

**Table 1:** Comparative Analysis of Nanomaterials in SPR Sensing: Performance, Stability, and Limitations.

Material Class	Enhancement Mechanism	Sensitivity Potential	Environmental Stability (Oxidation/Thermal)	Cost	Primary Limitation
AuNPs (0D)	LSPR field amplification	Medium	High (Inert)	High	Fixed resonance peak; limited field confinement compared to anisotropic shapes.
AgNPs (0D)	Strong LSPR & scattering	High	Low (Prone to oxidation)	Medium	Poor chemical stability in physiological buffers requires passivation layers (SiO_2_/Graphene).
1D/2D Noble Metals	Tunable LSPR & Hot spots	Very High	Medium/High (depends on capping)	High	Complex synthesis; challenging to fabricate uniform large-area arrays for reproducible sensing.
Graphene	Charge transfer & Surface area	Medium	High	Medium	Zero bandgap limits optical absorption; primarily serves as a functionalization interface rather than a plasmonic exciter.
MXenes	Tunable optoelectronic properties	High	Low/Medium (Oxidizes in water)	Medium	Prone to degradation in aqueous solutions; synthesis maturity is currently lower than graphene.
MNPs (Fe_3_O_4_)	Magnetic enrichment	N/A *	High	Low	Mainly used for analyte separation/enrichment; offers minimal direct SPR enhancement without metallic coating.

*: Fe_3_O_4_ acts as a carrier for analyte separation and pre-concentration but serves no function as a direct optical transducer for SPR signal amplification.

**Table 2:** Summary of Key Performance Indicators and Clinical Translation of Nanomaterial-Enhanced SPR Sensors.

Research Direction	Core Technology/Platform	Type of Performance Indicator	Representative Quantitative Breakthrough	Key Advantages
Intelligent Design Optimization	PCF-SPR/XAI	Maximum Wavelength Sensitivity	Beyond traditional limits; millisecond-level design	Design acceleration; parameter interpretability
Trace Biomarker Detection	SPR/SERS Dual-Mode, RBCM-AuNP System	Limit of Detection (LOD)	fM-level	Ultra-trace detection; high selectivity
Clinical Multiplex Diagnosis	SPRi Multi-Array	Average Accuracy in Real Samples	≥95%	Real-time; simultaneous multi-marker detection
On-Site Rapid Detection	LFS-POC/Microfluidics	Sample-to-Result Time/Accuracy	15 min/98% (SARS-CoV-2 detection)	Rapid; portable; automated
Environmental Pollutant Detection	SPR/SERS Dual-Mode, LRSPR-AgNP System	SERS LOD/Dynamic Range	nM-level LOD; 0.5–50 ppm (methylene blue)	Wide dynamic range; result cross-validation

## Data Availability

No new data were created or analyzed in this study.
